# Vitamin B12, folate and iron levels in primary nocturnal enuresis

**DOI:** 10.12669/pjms.311.6424

**Published:** 2015

**Authors:** Sebahattin Albayrak, Kürsad Zengin, Serhat Tanik, Ghaniya Daar, Mustafa Yasar Ozdamar, Hasan Bakirtas, M. Abdurrahim Imamoglu, Mesut Gurdal

**Affiliations:** 1 Sebahattin Albayrak, MD. Department of Urology, Bozok University, School of Medicine, Yozgat, Turkey.; 2 Kürsad Zengin, MD. Department of Urology, Bozok University, School of Medicine, Yozgat, Turkey.; 3 Serhat Tanik, MD. Department of Urology, Bozok University, School of Medicine, Yozgat, Turkey.; 4 Ghaniya Daar, MD. Bozok University, School of Medicine, Yozgat, Turkey.Department of Pediatrics,; 5 Mustafa Yasar Ozdamar, MD. Bozok University, School of Medicine, Yozgat, Turkey.Department of Pediatric Surgery; 6 Hasan Bakirtas, MD. Bozok University, School of Medicine, Yozgat, Turkey.; 7 M. Abdurrahim Imamoglu, MD. Bozok University, School of Medicine, Yozgat, Turkey.; 8 Mesut Gurdal, MD. Bozok University, School of Medicine, Yozgat, Turkey.

**Keywords:** Vitamin B12, Folate, Iron, Primary Nocturnal Enuresis

## Abstract

**Objective:**

Folate, vitamin B12 and iron are important vitamin and minerals which play role in the development of nervous system. The aim of this study was looking at the presence of folate, vitamin B12 and iron deficiency among patients with Primary nocturnal enuresis (PNE) and possible relation between the delay of central nervous system (CNS) development, PNE and folate, vitamin B12 and iron states.

**Methods:**

Consecutively applied forty patients with PNE (23 girls and 17 boys) and otherwise normal thirty control subjects (17 girls and 13 boys) were included in the study. Average ages (in range) of PNE and the control group were 9.2(6-12) years and 9.3 (6-12) years accordingly. Age, height, weight, complete blood count, blood vitamin B12, folate, ferritin and iron values of both groups were recorded and compared to each other.

**Results:**

Average vitamin B12 and folate levels of patients with PNE were significantly and statistically lower compared to those of the control group. Average blood iron of patients with PNE was significantly higher than that of the control group and also average ferritin level of the PNE group was detected to be higher than the control group but this relation was statistically insignificant.

**Conclusion:**

Primary nocturnal enuresis is related to the delay in CNS maturation so it was thought that low vitamin B12 and folate which were found in patients with PNE may have role in the delay of CNS maturation. Additionally, further studies are needed to investigate the role of vitamin B12 and folate either alone or as combination in treatment of patients with PNE who have low vitamin B12and folate level.

## INTRODUCTION

According to The International Children’s Continence Society (ICCS), enuresis has been described as normal voiding that occurs at an unsuitable and socially intolerable time and place.^        1 ^ Primary nocturnal enuresis (PNE) is a common finding in school-age children, with a prevalence rate around 9% in children between 5 and 10 years of age.^   2 ^

Conservative approach can be considered for monosymptomatic nocturnal enuresis as the first level of treatment. A successful urotherapy can save a patient from excessive medical interventions and other alternative treatment options.^3^ Biofeedback method without drug usage which needs active parent and child participation can be alternative treatment modality due to its short-term satisfactory results in enuresis nocturna.^4^ ICCS endorses the use of desmopressin as a first-line treatment for primary nocturnal enuresis of polyuric origin (level 1, grade A).^5^

Although PNE is a common disorder with a wide in psychological influence on afflicted children and an economic load on the children’s families, the pathogenesis of enuresis is unclear. It was found that maturational delay of the central nervous system is a significant factor in the pathogenesis of PNE.^6^ Previous studies have reported that PNE is principally correlated with genetic factors, including insufficiencies in the secretion of arginine vasopressin, bladder dysfunction, and sleep awareness disorder, among other factors.^7^ However, the role of low vitamin B-12 status in cognitive decrease still remains unclear.^8^ The Yokusoglu et al. study shows a disturbed autonomic balance in patients with true iron deficiency anemia (IDA).^9^ Rodent models demonstrate that iron deficiency during gestation/lactation changes myelination, neurotransmitters, neurometabolism, and gene/protein profiles before and after iron repletion at delectation. Human infants with IDA test lower in social-emotional, neurophysiologic, cognitive, and motor development than collation group infants.^10^ Vitamin B12 and folate are among important vitamins playing role in the development of nervous system.

In this study, our aim was to compare vitamin B12, folate and iron levels of patients with PNE and otherwise normal subjects. Also we aimed at searching the presence of folate, vitamin B12 and iron deficiency among patients with PNE and possible relation between delay in central nervous system (CNS) development, PNE and folate, vitamin B12 and iron states. 

## METHODS

Forty PNE patients and 30 otherwise healthy subjects who consecutively applied to pediatrics or urology clinics of Bozok University Research Hospital were included in the study. PNE was identified as bed-wetting in a child who never had a dry period for over 6 months according to the International Children Continence Society recommendations.^11^ Patients inclusion criteria included: a frequency of two or more enuretic episodes per week, not related to day time wetting, no dry period of more than 3-month duration since birth, normal pubertal stage for age evaluated by the Tanner staging scale.^12^ The study was approved by the local ethics committee of Bozok University (604/20-2014).

Detailed physical examination and medical records including age, weight, and height of all patients were obtained accordingly. Patients with any chronic disease, neurologic or urologic anomalies, previous urinary tract infection were excluded. All subjects in the study had sterile urinary culture and normal urinary analysis. Subjects had normal blood biochemistry and complete blood count. Vitamin B12, folate, ferritin and iron levels were measured. Serum folic acid levels were defined as follows: serum concentration below<3 ng/ml for deficiency, 3–6 ng/mL for low levels, and >6 ng/mL for normal values. The cutoff points used for vitamin B12 was 200 pg/mL, normal range 200 to 900 pg/mL, and excess>900 pg/mL. The cutoff points used for iron 40µg/dL, normal range40 to 150µg/dL, and excess>150 µg/dL. The cutoff points used for ferritin 4 ng/mL, normal range4 to 200 ng/mL, and excess>200 ng/mL.

Age, height, weight, complete blood count, blood vitamin B12, folate, ferritin and iron values of both PNE and control groups were compared to each other.

***Statistical Analysis: ***Shapiro-Wilk’s and Levene’s tests were used to test the normality and variance homogeneity of the data. Values were expressed as frequencies and percentages, mean ± Standard deviation or median and 25th-75th percentiles. To compare parametric continuous variables, Student's t-test was used. Age, height, weight, complete blood count, blood vitamin B12, folate, ferritin and iron values of both PNE and control groups were analyzed by using student’s t test. Statistical analyses were performed using the statistical package SPSS, version 15.0 (SPSS Inc., Chicago IL, USA); a value of P<0.05 was used to define statistical significance.

## RESULTS

Consecutively applied forty patients with PNE (23 girls and 17 boys) and otherwise normal thirty control subjects (17 girls and 13 boys) were included in the study. Average ages (in range) of PNE and the control group were 9.2 (6-12) years and 9.3 (6-12) years accordingly. Age, height, weight, hemoglobin and hematocrit values of both groups were statistically similar (Table-I). Average vitamin B12 value of PNE group (396 ± 97 pg/mL) was significantly lower than that of the control group (469 ± 82 pg/mL) (p=0.01). Similarly average folate value of PNE group (10.1 ± 2.2 ng/mL) was significantly lower compared to the control group (12.6 ± 3.5ng/mL) (p=0.03). Average blood iron value of PNE group (95.2 ± 20 µg/dL) was significantly higher than that of the control group (76.9 ± 21 µg/dL) (p=0.04). It was found that average blood ferritin value of PNE group (25 ± 5ng/mL) was higher than that of the control group (22.2 ± 4.2ng/mL) but this wasn’t statistically significant (p=0.45)(Table-II).

## DISCUSSION

In our study, it was found that vitamin B12 and folate needed for CNS maturation was lower among patients with PNE compared to the control group. These results were in parallel to the literature and it implies the role of retardation of CNS maturation in pathogenesis of PNE and it suggests possible relation between low vitamin B12 and folate, and delay in CNS maturation. To our knowledge, there is no yet reported study in the literature published in English on the subject of vitamin B12, folate, and iron among patients with PNE. Therefore, this study is first in this field.

The pathogenesis of PNE has not yet clearly been explained, but retardation in CNS maturation is thought to be an important factor in the pathogenesis.^13^^-^^15^ There are several theories proposed for the possible reasons of enuresis. Some of those theories are related to retardation in CNS maturation, psychogenic and behavioral components, environmental factors, allergens, low urinary bladder capacity, deep sleep disorder, structural anomalies of urinary tract, uninhibited neurogenic contractions, defects in diurnal secretion of vasopressin, prostaglandin production disorders and sleep apnea syndrome.^16^^,^^17^ Retardation in the growth rate, decreased bone age and bone mineral density identified in children with nocturnal enuresis may reflect delayed maturation of regulatory central nervous system functions.^18^ It is a well known fact that vitamin B12 deficiency can result in neurological diseases. Vitamin B12 deficiency especially effects hematopoietic, epithelial and nervous tissues but its role in nervous system metabolism is not totally clear.^19^ In case of vitamin B12 deficiency, hematologic, dermatologic, mucous membranes and nervous systems are effected. In nervous system, especially peripheric nerves, optic nerve, posterior and lateral columns of spinal cord and brainstem are damaged. B12 deficiency can result in wide-range of sign and symptoms from neural tube defects to cognitive and behavioral changes.^20^ In our study, it was found that average vitamin B12 value of PNE group was significantly lower than that of the control group (p=0.01). Low vitamin B12 values may retard CNS maturation and may lead other unknown pathologies which may be related to the pathogenesis of PNE. 

Folate is an essential vitamin for CNS development. Insufficient folate activity during pregnancy can result in neural tube defects. Folate is especially important for early stages of brain development. Folate deficiency can cause megaloblastic anemia, congestive heart failure, pigmentation, early graying of the hair, infertility, cervical dysplasia, endometrial dysplasia, and neuropathy, and dementia, psychiatric and cognitive disorders.^21^ In our study, it was found that average folate value in the PNE group was significantly lower compared to the control group. Low folate level similar to low vitamin B12, suggested that folate may also play role in CNS maturation. Additionally, it may be possible that CNS pathologies due to low folate play role in the development of PNE.

Deterioration of autonomic functions in patients with anemia due to vitamin B12 deficiency, sickle cell anemia and thalassemia major has been reported, but we do not have enough knowledge about the level of autonomic functions in iron deficiency which is leading cause of anemia. Yokusoglu et al. reported increased parasympathetic activity in case of iron deficiency anemia.^8^ In an animal study, siblings of animal with gestational iron deficiency revealed several disorders in the process of central nervous system development.^22^ Both acute and chronic malnutrition including iron and iodine deficiencies clearly show damage to the brain development.^23^ Previous studies have reported that iron deficiency similar to the vitamin B12 and folate deficiencies effects the brain maturation negatively. Iron and ferritin levels were expected to be lower among patients with PNE as in vitamin B12 and folate, but in reverse we found that average blood iron value of PNE group was significantly higher than that of the control group. Average blood ferritin value in PNE group was higher than that of the control group but this was not statistically significant. Although they had higher iron and ferritin levels, we could not get clear information whether they experienced such deficiency during early ages or gestational period. In our patients, CNS maturation may be disordered due to iron deficiency during early ages or gestational period.

**Table-I:** Baseline Characteristics of Participants (n = 70).

**Variables**	**PNE group (n = 40)**	**Control group (n = 30)**	**P value**
Age (year)	9.2 ± 2	9.3 ± 2	0.712
Height (cm)	122 ± 14	127 ± 15	0.211
Weight (kg)	27.5 ± 7.2	28.3 ± 7.9	0.585
Hemoglobin (gr/dl)	12.94± 1.26	12.81± 0.79	0.650
Hematocrit (%)	39.2 ± 3.61	38.1 ± 3.23	0.585

Not significant (P > 0.05).

**Table-II:** The comparison of vitamin B12, Folate, Iron, Ferritin levels of Primary nocturnal enuresis and the control groups

**Variables**	**PNE group (n = 40)**	**Control group (n = 30)**	**P value**
Mean vitamin B12 level (pg/mL)	396.4± 117.2	469.8± 125.2	**0.099**
Mean Folate level (ng/mL)	10.1 ± 2.1	12.6 ± 2.2	**0.035**
Mean Iron level (ng/mL)	95.2 ± 21.2	76.9 ± 20.4	**0.041**
Mean Ferritin level (ng/mL)	25.1 ± 4.1	22.2 ± 3.8	0.455

Significant (P < 0.05)

## CONCLUSION

It was suggested that low vitamin B12 and folate which were found in patients with PNE may have role in delay of CNS maturation. Additionally, further larger studies are needed to investigate the role of vitamin B12 and folate either alone or in combination may be used in the treatment of patients with PNE who have low vitamin B12 and folate level. In this respect, our study is pioneering.

## Notes

***Source of funding: ***All authors declare no source of funding.
